# Teaching Facilitation of Family Participation in Educational Institutions

**DOI:** 10.3389/fpsyg.2021.748710

**Published:** 2022-02-18

**Authors:** María Ángeles Gomariz, Joaquín Parra, María Paz García-Sanz, María Ángeles Hernández-Prados

**Affiliations:** ^1^Department Research Methods and Diagnostic in Education, Faculty of Education, University of Murcia, Murcia, Spain; ^2^Department Theory and History of Education, Faculty of Education, University of Murcia, Murcia, Spain

**Keywords:** parent participation, teacher, questionnaire, validity, reliability

## Abstract

The participation of families in schools where their children study is a recurring research topic. This field tends to address the perception of parents or teaching staff. This work is novel in that it considers what teachers, and not families, do to facilitate this participation. The purpose of this work has been to contrast a theoretical model with a multidimensional questionnaire designed to obtain information on the assistance provided by teachers to improve parental involvement in schools. It will allow us to lay the foundations for the content necessary for the initial and permanent training of teachers. Then, an initial questionnaire was created and, after being subjected to expert judgment, it was applied to 225 Spanish teachers, using a quantitative and a non-experimental methodology. After calculating the exploratory and confirmatory factor analysis and applying the structural equation model, a questionnaire (QFIS-TP) was obtained that had satisfactory construct validity and reliability.

## Introduction

The socioeconomic development of a country is closely tied to its education and ability to generate scientific-technological knowledge and innovations (Spanish Strategy of Science and Technology and of Innovation [EECTI], 2012). Improved educational quality depends not only on economic issues (Organisation for Economic Co-operation and Development [OECD], 2012, 2014), but also on the modernization of systems, through the development of associations and unions for innovation, to capitalize on existing resources (Europe Strategy 2020, 2015). It involves structural and functional changes that affect the school community, requesting the promotion of communication and participatory channels, especially between families and teachers.

Most countries in the European Union have taken on the challenge of promoting school democratization, reassessing the channels of family participation (European Commission, 2005) as a useful measure in response to scholastic failure and student dropout (Organisation for Economic Co-operation and Development [OECD], 2011), and opting for assessment processes that collect student and parent opinions on their educational implication (Maxwell and Staring, 2018). Ultimately, it has been recognized that parents are critical and demanding consumers of education who can contribute to its improvement through collaborative efforts, or to its decline through their passivity or destructive criticism (European Commission, 2000). In this dichotomy, the prevailing position depends on how family participation is understood, as well as the dynamics promoted by the teaching staff. So, the decision to participate depends on the feeling that one is welcomed by the school, children, and teachers, who utilize various means to express to parents that their participation is necessary and useful (Walker et al., 2005). But, in what content should teachers be trained to promote the participation of families in school?

In addition to the knowledge of degrees of family participation, the complexity of the question requires the creation of a theoretically solid instrument that allows measuring how teachers promote this participation. Although necessary, few studies have analyzed teachers as mediators or promoters of this participation (Erdener, 2016; Gomariz et al., 2017; Yulianti et al., 2019). This is the focal point of this work.

### Teachers as Promoters of Family Participation

The main research question guiding the bibliographic review was focused on the role teachers play in family participation in the educational institutions. The lack of scientific research on this topic prevents the identification of the teacher’s role in facilitating participation, limiting the search to relevant theoretical models, reviews, and meta-analyses from the past two decades. So, various theoretical models of family participation have been found. The model of Epstein (2011), which distinguishes between six modalities of family participation (parenting, communicating, volunteering, learning at home, decision making and collaborating with community); that of Vogels (2002), which considers four family profiles related to scholastic education (consumers, clients, participants and partners); the renewed model of Hoover-Dempsey and Sandler (Walker et al., 2005), focusing on the aspects that explain why parents get involved and how their participation improves the scholastic performance of their children (involvement decision, forms, influence, mediating variables and student outcomes); Hornby (2011), which includes eight types of family participation (communication, liaison, education, support, information, collaboration, resource, and policy) and that of Garbacz et al. (2019), which considers five participation dimensions (communication, expectations, educational support, community activities, and scholastic assistance). All of these provide a theoretical foundation to form the core of the Integral Model of Family Involvement in School–Teacher Promoter (IMFIS-TP), which we propose and detail below and which includes 7 dimensions. It is a new model that focuses on the teacher figure as the driving force behind specific actions that favor family participation in schools, and not only attributions or good intentions, since expressing the desire and need for increased participation does not contribute to making this participation a reality.

### Teacher–Family Communication

The teacher’s responsibility in the family communication process has been widely recognized, understanding that parent–teacher tutorials tend to be initiated upon request of the teachers, and less frequently, upon request of the families (Epstein, 2011; Tran, 2014). These communicative meetings offer unique opportunities for the “teacher-promoter” to foster the child’s educational support from the home (Dettmers et al., 2019) and the families’ sense of belonging to the center (Tran, 2014).

Parents need to understand diverse aspects of their children’s’ schooling and must feel that they are free to communicate *via* diverse channels and at any time. Therefore, teachers should offer families a wide range of communication channels (Hornby, 2011). The use of agendas, attitude toward participation, the language used, flexibilization of meeting schedules, and the use of the social networks all help to increase family participation, assuming the appropriate use of the same (Erdener, 2016).

### School Activities

This dimension focuses on the promotion of collaborative school–family actions in the educational institution, although in combination with activities taking place outside of the school and training activities (community participation and family training, which we will discuss further in this work). Families act as volunteers in a wide variety of activities (school patrolling, student transport, classroom workshops, sporting activities, scholastic camp, fund collection, etc.) which are promoted and coordinated by teachers (Epstein et al., 2019) using distinct participation modalities, such as attendance, collaboration, or involvement in management and decision making (Consejo Escolar del Estado, 2014).

### Sense of Belonging

Although research has demonstrated that the sense of belonging to a school is the driving force behind participation (Uslu and Gizir, 2017), few studies have analyzed how teachers contribute to fostering this participation by the families. Reference has been made to the sense of belonging and recognition by the educational community and how this can be promoted through a greater knowledge of the school, trust in the teaching staff, removal of myths and prejudices, meetings with families, satisfaction with the school activities carried out, etc. (Cheung et al., 2017). It is also related to positive communication with teachers (Anderson and Minke, 2007) and involvement in the home (Uslu and Gizir, 2017). Indeed, “Whereas most parents are reluctant to seek the help of professional counselors, they will approach their children’s teachers in search of guidance or counseling for the problems that concern them” (Hornby, 2011, p. 37).

### Involvement in the Home

The expression “family involvement” refers, on the one hand, to extra-scholastic, cultural, family leisure, and value-transmission activities (Consejo Escolar del Estado, 2014; Caldas and Cornigans, 2015) and, on the other hand, to parent expectations, homework supervision, promoting reading, and reserving the term “participation” to refer to school activities (Castro et al., 2015). Parental support in the home relies on teacher counseling to redirect and encourage the children’s studies (Kurtulmus, 2016). It may be offered on an individual or a group manner, and requires the creation of effective, trust-based communicative processes (Hornby, 2011). All of this contributes to the creation of a sense of belonging to the community.

### AMPA (Parent Association) and School Board

The legal model of joint responsibility regulated *via* the parent association (PA) and School Board contributes to improved performance; family participation; educational quality; and knowledge of citizen rights, responsibilities, and duties (Benner et al., 2016). It promotes aspects related to the school and the community, and its success is based in large part on collaboration, recognition, and dissemination of the same by the teacher (García-Sanz et al., 2020). However, although this model is quite extensive and has reached most schools, only a small minority of parents are actively involved (Hornby, 2011), usually as associates and not as managers, be it in the PA management team or as a School Board representative (García-Sanz et al., 2020).

### Community Participation

Teachers, in collaboration with the PA and in support of a bidirectional relationship between schools and society, should know how to involve their students’ families in cooperation with community/neighborhood organizations. This involves the use of external (libraries, parks, museums, etc.), leisure, volunteer, and solidarity action services, religious events, community associations, or strength–talent training (Epstein et al., 2019; Gahwaji, 2019; Garbacz et al., 2019). According to Severiens et al. (2014), these activities should be included in teacher training curriculums since community projects are more beneficial when they are directed at the entire school population, as opposed to those that are individualized and only from the school.

### Family Training

Parental education helps contribute to school improvement. This school-based activity is often managed by PAs and tends to be directed at families, although recently, community training has also been promoted, with parents and teachers learning together (Tran, 2014; Hernández-Prados, 2019). This explains the close association between training, school activities, the PA–School Board, and community participation, since they often rely on municipal training offers or external professionals, especially in the case of public schools (Hernández-Prados et al., 2019).

Using the IMFIS-TP as a foundation, the following research question was proposed: How is it possible to validly and reliably assess the way and the degree to which teachers facilitate family participation in their children’s schools? The purpose of the study is to create a multidimensional questionnaire directed at teachers that is based on the IMFIS-TP, which allows us to obtain information regarding the assistance offered by teachers so that parents may participate in the distinct dimensions making up family participation in education institutions. The questionnaire will be very useful to know where to direct the initial and continuous training of teachers.

## Materials and Methods

### Participants

The invited population consisted of 542 teachers from 14 multicultural educational institutes in southeastern Murcia (Spain), where they teach Early Childhood, Primary, and Secondary Education. Of these, 225 teachers agreed to participate, resulting in a 95% confidence level and a 5% sampling error.

### Instrument

Based on the IMFIS-TP, an initial questionnaire was created with 11 socio-demographic questions and 91 items on teacher facilitation of family participation, grouped into seven dimensions: “Questionnaire on Family Involvement in School, Teacher-Promoter” (QFIS-TP). After performing an interjudge content validation with 5 university professors (experts in family-school relations and methodology) and the 14 management teams from the participating schools, the instrument kept the 11 socio-demographic questions, but the family participation items were reduced to 74 (Table 1).

**TABLE 1:** QFIS-TP dimensions and items.

**A.Communication with the center** **Use an X to mark the frequency or degree to which, in your opinion, the content of each of the questions below arises, based on the indicated scale.**
(1) I inform families of the importance of attending teacher-parent conferences when they are requested.
(2) I promote family requests of teacher-parent conferences throughout the school year.
(3) I urge families to attend group meetings of parents with the teacher, which are convened by the teaching staff.
(4) I encourage families, at least, to speak with the teacher in casual meetings at the entrance or exit of the school.
(5) I encourage families to hold meetings with the other teachers in the school, apart from their child’s main teacher.
(6) In general terms, the school facilitates communication with the students’ parents.
**B.Participation in school activities** **Indicate how you facilitate family involvement in the following activities, only in the cases in which they are organized by their child’s educational institution, based on the indicated scale.**
(7) In workshops in the classroom (on reading, crafts, cooking, etc.)
(8) In cultural activities (historic events, musical and ecological topics, traditions, international day of peace, grandparent’s day, children’s day, women’s day, etc.)
(9) In sporting activities (soccer, basketball, judo, karate, etc. camps or exhibitions).
(10 At celebrations (Christmas, Carnival, end of year, etc.).
(11) In outings (to museums, monuments, other institutions, field trips, etc.).
(12) In service activities offered by the school (receiving children in classrooms before the school day begins, library, cafeteria, student transport, etc.)
(13) In work commissions created in the school (co-existence plan, school improvement plan, etc.)
(14) In economic collection commissions for classroom (gifts, costumes, classroom decorations, etc.)
(15) In processes used to assess the school (responding to questionnaires, using the suggestions box, presenting complaints and/or suggestions via PA or individually, etc.)
(16) In general, the school promotes family participation in the activities that it organizes.
**C.Sense of belonging** **Indicate your degree of agreement or disagreement with the following Statements, based on the indicated scale.**
(17) I urge families to identify with the school’s values, ideas, attitudes, goals, etc.
(18) I encourage families to feel that they are members of the school, so that they consider it to be their own.
(19) I promote the ideas in families that when a sporting, artistic or cultural team participates in a championship, contest or exhibition, it is their team.
(20) I talk with families to promote their trust in the educational work being carried out by teachers with their children, encouraging support of our decisions.
(21) I encourage families to feel attracted by the collaborative activities or experiences offered by the school.
(22) I help families perceive that their participation in the school makes them a part of the same.
(23) I encourage families to feel welcomed and integrated in the school community as of the onset of their child’s schooling.
(24) I do everything possible to ensure that families feel satisfied with the education that the students receive.
(25) I guide families so that they feel free and can express their ideas, concern, suggestions, complaints, etc. in the school.
(26) I encourage families to recommend this school to others with school-aged children.
(27) Generally speaking, the school assists in creating a bond between it and families.
**D.Involvement in the home** **Mark the frequency or degree to which, in your opinion, the content of the statements presented below takes place, based on the indicated scale.**
(28) I guide parents in speaking with their children about what they do in class.
(29) I encourage families to express their trust in their children.
(30) I inform families of their child’s class attendance.
(31) I encourage families to take an interest in the educational tasks carried out by their children at home.
(32) I inform families of the importance of their children’s study time organization.
(33) I encourage families to offer a good at-home study climate (to encourage studying, offer an appropriate study site without distractions, provide resources for learning, etc.)
(34) I inform families of the need to demonstrate their availability to their child’s needs regarding their school work.
(35) I invite parents to congratulate their children when they complete their school work.
(36) I notify families of the importance that their children complete extra-curricular or complementary activities (languages, computer-based, music, dance, sports, academic activities, etc.)
(37) I communicate with families to promote the autonomy and responsibility of their children in their studies (encouraging them to be alert, but not to do their child’s work or always be next to them when doing it).
(38) I speak to families so that they oversee the responsible use of computers, mobile phones, etc.
(39) I encourage families to engage in cultural activities (read, go to the cinema, theater, museums, trips, concerts, exhibitions, etc.)
(40) I encourage families to ensure that their children apply what they learn in school to their everyday lives.
(41) In general, the school facilitates parent involvement at home in the educational process of their children.
**E.Involvement in the PA and the School Board** **Mark the frequency or degree to which, in your opinion, the content of the following statements takes place, based on the indicated scale.**
(42) I speak with families so that they are informed with regard to the organization and functioning of the PA.
(43) I encourage families to know the members of the PA Board of Directors.
(44) I help families gain knowledge regarding the activities organized by the PA.
(45) I speak with parents so that they are informed and use the municipal bank of books in which the PA participates.
(46) I encourage families to consult information on the PA via websites, the social networks, etc.
(47) I invite families to participate in activities organized by the PA.
(48) I encourage families to form a part of the school’s PA Board of Directors.
(49) I inform the PA of the importance that the association represents the interests of all of the school’s families.
(50) Generally speaking, the school promotes parent participation in the PA.
(51) I help families to get to know the organization and functioning of the School Board.
(52) I encourage families to get to know their School Board representative.
(53) I invite families to be informed of the decisions made in School Board meetings.
(54) In encourage families to be informed as to the elections process of the School Board (calendar, candidates, voting procedure, etc.).
(55) I encourage families to participate in School Board elections.
(56) I encourage parents to apply for family representative positions in School Board elections.
(57) In general, the school promotes family participation in the School Board.
**F.Community participation** **Indicate how you facilitate family involvement in the following activities related to the community, based on the indicated scale.**
(58) In collection activities (collection of food, clothes, caps, solidarity markets, etc.)
(59) In ecological activities (cleaning of waterways, march in support of the environment, environmental awareness programs, tree planting, etc.)
(60) In activities organized by neighborhood associations (block parties, neighborhood meetings, neighborhood or city needs, presence in the neighborhood councils, etc.)
(61) In solidarity and volunteering activities (assistance to the elderly, the sick, those with limited resources, those who are alone, soup kitchens, etc.).
(62) In activities of the distinct religious communities.
(63) In activities directed at diversity awareness and integration (gender, abilities, cultural, ethnic background, etc.).
(64) In collaboration activities with youth associations promoting healthy leisure and free time.
(65) In general, the school promotes family participation in community activities.
**G.Training** **Mark the frequency or degree to which, in your opinion, the content of each of the statements below arises, based on the indicated scale.**
(66) I guarantee that the families are informed of the training activities directed at the same that are organized by the education institution.
(67) I encourage families to attend training activities organized by the school.
(68) I speak with families to encourage their participation in training activities that are intended for them.
(69) I encourage parents to get involved in the management of training activities for families.
(70) I notify families of the importance of receiving appropriate training with regard to their children’s educational process.
(71) I relate the training that is being offered by the school to an improvement in family-school relations.
(72) Generally speaking, the school facilitates participation in training activities intended for families.
(73) I need training in order to better facilitate family involvement in the education of their children.
(74) I am interested in attending training activities to improve family participation in the education of their children.

The scale of items contains 5 degrees, except for item 74, which is dichotomous (Yes/No). In the following dimensions, namely communication with the school, involvement in the home, participation in the PA and school board, training, and all related to school facilitation for…, interpretation of the scale is: 1. Never/none; 2. Infrequently/few; 3. Sometimes/somewhat; 4. Often/considerably; 5. A lot/many. In the dimensions of Participation in school activities and Community participation, the scale ranges from: 1. I don’t know if they are carried out; 2. I know of them; 3. I attend; 4. I collaborate, participating; 5. I am involved in their organization. Also, those referring to the Sense of belonging dimension are responded to using the following scale: 1. Completely disagree; 2. Disagree; 3. Indifferent; 4. Agree; 5. Completely agree.

### Procedure

The validity of the content of the initial QFIS-TP was determined by sending an email to two types of judges (university educators and school management teams), permitting the modification of any items and elimination of others, when considered to be repetitive.

Upon validation, the QFIS-TP was applied to the professors on-line, using the survey platform of the University of Murcia. The research method was quantitative, non-experimental, descriptive and transversal, survey-type. Next, the data were analyzed using the IBM SPSS, v. 24 and IBM SPSS Amos, v.21 programs.

## Analysis and Results

### Exploratory Factorial Analysis

To calculate the exploratory factorial analysis (EFA) using the main components extraction and Varimax rotation methods, the SPSS program was used, but first, the Spearman’s correlation coefficient was obtained between all items of the instrument to avoid problems of multicollinearity. No bivariate correlations exceeding 0.85 were found; therefore, it was not necessary to eliminate any item (Kline, 2005). In addition, the Kaiser–Meyer–Olkin (KMO) measure of sampling adequacy of 0.928 and the statistical significance of the Bartlett sphericity test (0.000) permit the EFA.

Coinciding with the dimensions of the IMFIS-TP, seven components were determined, having an explained variance of 64.70%. This initial EFA consisted of all of the ordinal items of the questionnaire, except for those related to the teacher’s perception of what the school does to facilitate family participation. With these items, a second EFA was created, since it was considered that its content did not specifically address teacher’s attitudes and behaviors to collaborate in the improvement of family participation. Table 2 shows the rotated components matrix, ordered by size, referring to the first EFA.

**TABLE 2:** First EFA: rotated components matrix.

Items	Component						
	1	2	3	4	5	6	7
Q51	**0.862**	0.076	0.089	0.169	0.103	0.167	0.094
Q55	**0.861**	0.032	0.156	0.142	0.144	0.135	0.068
Q54	**0.856**	0.065	0.173	0.151	0.170	0.170	0.063
Q56	**0.843**	0.068	0.132	0.170	0.200	0.174	0.023
Q52	**0.832**	0.063	0.088	0.193	0.207	0.196	0.006
Q48	**0.829**	0.132	0.093	0.165	0.215	0.178	0.019
Q53	**0.798**	0.133	0.142	0.203	0.174	0.214	0.026
Q42	**0.772**	0.241	0.083	0.105	0.195	0.080	0.179
Q43	**0.771**	0.219	0.082	0.163	0.219	0.195	0.004
Q49	**0.768**	0.083	0.133	0.233	0.266	0.075	−0.010
Q47	**0.768**	0.217	0.125	0.170	0.114	0.094	0.143
Q44	**0.754**	0.235	0.100	0.072	0.262	0.134	0.174
Q46	**0.709**	0.270	0.078	0.265	0.223	0.091	0.064
Q45	**0.634**	0.267	0.105	0.187	0.037	0.134	0.236
Q34	0.115	**0.773**	0.146	0.126	0.070	0.148	0.065
Q33	0.169	**0.797**	0.137	0.065	−0.041	0.077	0.127
Q32	0.229	**0.759**	0.166	0.036	0.035	0.102	0.220
Q40	0.086	**0.757**	0.189	0.124	0.196	0.216	−0.044
Q37	0.102	**0.718**	0.177	0.053	0.057	0.090	0.302
Q31	0.153	**0.696**	0.151	−0.075	0.136	0.110	0.071
Q38	0.241	**0.668**	0.164	0.099	0.088	0.025	0.171
Q28	0.054	**0.658**	0.266	0.080	0.315	0.141	0.124
Q29	0.013	**0.608**	0.347	0.107	0.246	0.283	0.072
Q35	0.036	**0.572**	0.151	0.151	0.074	0.382	0.207
Q39	0.264	**0.534**	0.147	0.106	0.299	0.291	−0.065
Q30	0.176	**0.451**	0.157	0.085	0.002	−0.013	0.130
Q36	0.165	**0.354**	0.062	0.219	0.268	0.314	−0.043
Q24	−0.045	0.191	**0.798**	0.010	0.006	0.020	0.010
Q22	0.191	0.134	**0.767**	0.089	0.264	0.129	0.037
Q21	0.131	0.172	**0.764**	0.099	0.281	0.169	0.061
Q23	0.143	0.149	**0.762**	0.164	0.119	0.151	0.109
Q18	0.144	0.213	**0.761**	0.099	0.218	0.103	0.095
Q20	0.055	0.321	**0.707**	−0.008	0.078	0.013	0.092
Q17	0.170	0.332	**0.671**	0.071	0.115	0.137	0.057
Q25	0.212	0.289	**0.661**	0.062	0.113	0.076	0.149
Q26	0.290	0.058	**0.497**	0.216	0.065	0.225	0.049
Q19	0.204	0.026	**0.421**	0.216	0.269	0.322	0.074
Q61	0.242	0.093	0.085	**0.820**	0.136	0.083	0.017
Q59	0.170	0.080	0.041	**0.787**	0.185	0.151	0.096
Q63	0.211	0.106	0.162	**0.778**	0.092	0.196	0.061
Q60	0.328	0.072	0.069	**0.771**	0.232	0.087	0.035
Q62	0.296	0.020	0.079	**0.734**	0.153	0.006	−0.077
Q64	0.338	0.191	0.161	**0.678**	0.207	0.185	0.016
Q58	0.133	0.135	0.140	**0.531**	0.178	0.248	0.268
Q10	0.131	0.110	0.144	0.068	**0.720**	0.115	0.109
Q8	0.310	0.042	0.146	0.099	**0.683**	0.131	0.119
Q9	0.296	−0.003	0.078	0.198	**0.658**	0.108	0.165
Q11	0.218	0.215	0.032	0.262	**0.636**	0.137	−0.168
Q7	0.164	0.059	0.134	0.053	**0.603**	−0.005	0.162
Q12	0.270	0.188	0.104	0.235	**0.589**	0.074	0.034
Q14	0.140	0.089	0.241	0.107	**0.532**	0.028	0.080
Q15	0.214	0.128	0.185	0.169	**0.523**	0.169	0.074
Q13	0.258	0.188	0.165	0.317	**0.507**	0.162	0.027
Q68	0.393	0.262	0.206	0.147	0.141	**0.728**	0.094
Q67	0.336	0.259	0.267	0.170	0.182	**0.697**	0.179
Q69	0.402	0.218	0.104	0.218	0.285	**0.681**	0.075
Q70	0.292	0.281	0.170	0.220	0.183	**0.680**	0.114
Q71	0.324	0.218	0.215	0.207	0.179	**0.646**	0.137
Q66	0.375	0.269	0.197	0.166	0.051	**0.626**	0.227
Q73	0.243	0.152	0.097	0.044	0.044	**0.247**	−0.099
Q3	0.144	0.335	0.188	−0.013	0.158	−0.003	**0.703**
Q1	0.069	0.395	0.042	0.037	0.168	0.131	**0.673**
Q2	0.138	0.283	0.155	0.090	0.161	0.174	**0.617**
Q5	0.330	0.120	0.178	0.322	0.092	0.101	**0.525**
Q4	0.035	0.174	0.073	−0.030	0.275	0.158	**0.284**

*Bolded values are factor loadings of the items that make up each factor or component.*

As seen in Table 2, the first factor includes 14 items of the QFIS-TP dimension called involvement in the PA and School Board; the second 13 items are from the involvement in the home dimension; the third includes the 10 items corresponding to the sense of belonging dimension; the fourth consists of the 7 items from the community participation dimension; the fifth includes 9 items regarding the participation in school activities dimension; the sixth is made up of the 7 items from the training dimension; and the seventh and final factor includes the 5 items of the questionnaire regarding the communication with the school dimension.

Based on the results obtained in this EFA, it can be concluded that the QFIS-TP has not experienced any variations with regard to the assignment of items to each dimension in the content validation carried out by the experts and based on the premises of the IMFIS-TP. Therefore, the naming of the instrument’s dimensions, as well as that of the items making up each of these, has been maintained with no modifications.

The second EFA, based on items related to the school’s facilitation of families to improve the dimensions considered in the QFIS-TP, obtained a mean Kaiser–Meyer–Olkin sampling adequacy of 0.875 and a statistical significance according to the Bartlett’s Sphericity test of 0.000. The variance explained with Eigen values >1 was 55.95% (Table 3).

**TABLE 3:** Second EFA: components matrix.

Items	Component
	1
Q27	0.828
Q41	0.804
Q57	0.784
Q50	0.737
Q65	0.729
Q16	0.715
Q72	0.707
Q6	0.667

As we can observe, the eight items converge in one unique factor called: Facilitation of the school for family participation.

### Confirmatory Factorial Analysis

Using the AMOS program, the IMFIS-TP was corroborated. To do so, the construct validity of the QFIS-TP was ratified through the calculation of a confirmatory factorial analysis (CFA) using the structural equations model. To offer more sense to the theoretical model, the involvement in the PA and the School Board was separated into two dimensions. Similarly, missing values and outliers were eliminated, despite the fact that there was no agreement regarding the removal of the latter (Aguinis et al., 2013). Therefore, cases in which the standardized observable variables exceeded a score of | 3| were excluded (Verdugo et al., 2008).

**FIGURE 1:**
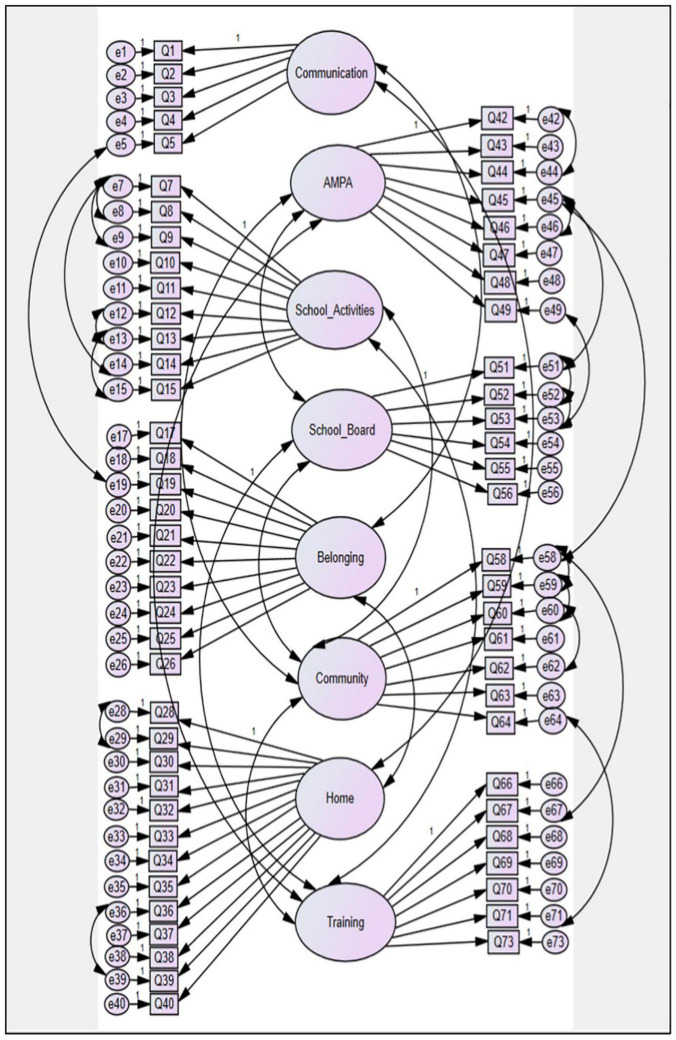
Structural equations model of the QFIS-TP.

In accordance with the IMFIS-TP, the correlation between latent and observable variables, the measurement error of the same, and the covariance between the latent variables and measurement errors are represented graphically in Figure 1.

The maximum likelihood method was used for model calculation, complying with the univariate normality criteria, having obtained values up to | 2| for asymmetry and up to | 7| for kurtosis (Curran et al., 1996).

Table 4 presents the regression coefficients (factorial loads) between the observable and latent variables, standard error (SE), critical ratio (CR), and the corresponding statistical significance (*p*). Likewise, it indicates the standardized regression coefficients between the observable and latent variables. It may be observed that all of the pairs are significant, with α = 0.01. Likewise, in all cases, the standardized regression coefficients exceed the typical value of the effect size (*r* ≥ 0.3), as determined by Cohen (1988).

**TABLE 4:** Regression coefficients and standardized regression coefficients between observable and latent variables.

Relationship between and latent variables		Regression weights				Standardized regression weights
		Estimate	SE	C.R.	P	Estimate
Q5	Communication	1.312	0.197	6.674	***	0.535
Q4	Communication	1.253	0.253	4.959	***	0.396
Q3	Communication	1.340	0.149	9.000	***	0.771
Q2	Communication	1.410	0.163	8.633	***	0.727
Q1	Communication	1.000				0.697
Q14	School_Activities	1.225	0.238	5.149	***	0.482
Q13	School_Activities	1.528	0.294	5.200	***	0.635
Q12	School_Activities	1.509	0.287	5.255	***	0.650
Q11	School_Activities	1.718	0.320	5.377	***	0.689
Q10	School_Activities	1.218	0.226	5.393	***	0.695
Q9	School_Activities	1.694	0.280	6.045	***	0.732
Q8	School_Activities	1.491	0.232	6.440	***	0.723
Q7	School_Activities	1.000				0.434
Q15	School_Activities	1.225	0.251	4.884	***	0.548
Q66	Training	1.000				0.775
Q67	Training	1.104	0.081	13.548	***	0.857
Q68	Training	1.290	0.092	13.961	***	0.893
Q69	Training	1.321	0.099	13.342	***	0.862
Q70	Training	1.099	0.090	12.248	***	0.806
Q71	Training	1.046	0.089	11.752	***	0.772
Q22	Belonging	1.340	0.126	10.598	***	0.835
Q21	Belonging	1.321	0.122	10.827	***	0.856
Q20	Belonging	0.994	0.115	8.633	***	0.666
Q19	Belonging	0.897	0.152	5.897	***	0.439
Q18	Belonging	1.076	0.113	9.510	***	0.740
Q17	Belonging	1.000				0.683
Q23	Belonging	1.339	0.128	10.439	***	0.821
Q24	Belonging	0.757	0.093	8.121	***	0.624
Q25	Belonging	1.024	0.120	8.535	***	0.658
Q26	Belonging	0.866	0.149	5.812	***	0.439
Q34	Home	1.020	0.108	9.423	***	0.759
Q33	Home	0.894	0.095	9.423	***	0.759
Q32	Home	0.894	0.092	9.700	***	0.786
Q31	Home	0.769	0.093	8.270	***	0.653
Q30	Home	0.733	0.103	7.128	***	0.553
Q29	Home	1.148	0.083	13.907	***	0.760
Q28	Home	1.000				0.663
Q35	Home	0.924	0.105	8.836	***	0.704
Q36	Home	0.941	0.151	6.244	***	0.480
Q37	Home	0.960	0.098	9.757	***	0.791
Q38	Home	1.061	0.122	8.687	***	0.690
Q39	Home	1.106	0.148	7.491	***	0.585
Q40	Home	1.235	0.134	9.226	***	0.740
Q45	AMPA	0.882	0.085	10.341	***	0.651
Q46	AMPA	1.068	0.080	13.393	***	0.786
Q44	AMPA	1.010	0.054	18.805	***	0.834
Q43	AMPA	1.133	0.072	15.718	***	0.866
Q42	AMPA	1.000				0.840
Q47	AMPA	1.188	0.070	16.868	***	0.901
Q48	AMPA	1.329	0.078	17.121	***	0.908
Q49	AMPA	1.262	0.085	14.842	***	0.837
Q53	School_Board	1.000	0.060	16.611	***	0.855
Q54	School_Board	1.152	0.051	22.418	***	0.974
Q52	School_Board	1.059	0.047	22.763	***	0.887
Q51	School_Board	1.000				0.869
Q55	School_Board	1.177	0.056	20.966	***	0.949
Q56	School_Board	1.191	0.057	20.904	***	0.948
Q61	Community	1.751	0.207	8.447	***	0.840
Q60	Community	1.530	0.188	8.125	***	0.786
Q59	Community	1.554	0.168	9.238	***	0.739
Q62	Community	1.378	0.182	7.563	***	0.701
Q63	Community	1.771	0.209	8.471	***	0.845
Q64	Community	1.611	0.195	8.274	***	0.795
Q58	Community	1.000				0.555
Q73	Training	0.466	0.105	4.445	***	0.323

*** Statistical significance of the regression between each item and the assigned construct.

Table 5 reveals the covariance coefficients between the latent variables and between the measurement errors of the observable variables, the SE, CR, statistical significance (*p*), and the respective standardized regression coefficients. As seen, the relationship between 84.62% of the pairs is significant at α = 0.01, whereas the relationship between the remaining 15.38% is statistically significant at α = 0.05. Likewise, the correlation coefficients reach (approximately) or exceed, in 79.49% of the cases, the typical value of the effect size (*r* ≥ 0.3) as established by Cohen (1988).

**TABLE 5:** Covariance and correlation between latent and observable variables.

Relationship between latent variables and between measurement errors		Covariance				Correlation
		**Estimate**	**SE**	**C.R.**	***P***	**Estimate**
AMPA	Community	0.264	0.056	4.735	***	0.449
Training	Community	0.233	0.049	4.756	***	0.484
School_Board	Community	0.275	0.062	4.450	***	0.397
School_Activities	Community	0.122	0.035	3.464	***	0.367
Training	AMPA	0.399	0.064	6.209	***	0.587
AMPA	School_Board	0.899	0.111	8.098	***	0.917
Training	School_Board	0.433	0.072	5.987	***	0.540
School_Activities	Training	0.113	0.033	3.392	***	0.292
Communication	Home	0.127	0.023	5.433	***	0.646
Communication	Belonging	0.108	0.020	5.313	***	0.607
Belonging	Home	0.149	0.027	5.603	***	0.653
e8	e7	0.305	0.065	4.700	***	0.386
e9	e7	0.222	0.066	3.351	***	0.254
e13	e12	0.281	0.075	3.749	***	0.321
e36	e39	0.193	0.051	3.760	***	0.291
e42	e44	0.138	0.031	4.416	***	0.384
e51	e52	0.139	0.028	4.968	***	0.382
e58	e59	0.256	0.065	3.931	***	0.289
e59	e60	0.144	0.049	2.928	0.003	0.203
e60	e62	0.332	0.063	5.257	***	0.475
e14	e7	0.279	0.084	3.324	***	0.226
e5	e19	0.188	0.052	3.574	***	0.277
e15	e13	0.156	0.069	2.246	0.025	0.168
e28	e29	0.155	0.025	6.138	***	0.557
e58	e45	0.121	0.055	2.182	0.029	0.133
e58	e67	0.189	0.038	4.929	***	0.395
e64	e71	0.139	0.043	3.249	0.001	0.273
e45	e51	0.135	0.038	3.530	***	0.236
e53	e52	0.120	0.028	4.249	***	0.310
e49	e53	0.083	0.036	2.313	0.021	0.169
e45	e46	0.167	0.054	3.116	0.002	0.233

*** Statistical significance of the regression between each item and the assigned construct.

In accordance with Hu and Bentler (1998), various index types have been used to assess the fit of the model: standardized Chi square or the relative Chi square over degrees of freedom (CMIN/DF), included in the measures of goodness of fit; comparative fit index (CFI), framed within the incremental fit measures; and the root mean square error of approximation (RMSEA), included in the absolute goodness of fit measures. Table 6 indicates the values obtained and those desired, according to the classification established by distinct authors (Hu and Bentler, 1998; Lévy and Varela, 2003; Hair et al., 2008; Cupani, 2012).

**TABLE 6:** Goodness of fit indices of the IMFIS-TP.

Index	Desired value	Obtained value
CMIN/DF	Between 1 and 5	1.88
CFI	≥0.9	0.90
RMSEA	<0.08	0.07

With these referents (Table 6), it may be affirmed that the IMFIS-TP has acceptable fit indices between the theoretical structure of the model and the empirical results obtained.

### Reliability of the Questionnaire

To obtain the reliability of the QFIS-TP, once again, the SPSS program was used. This psychometric property, both globally and by dimensions, was calculated using the Cronbach’s alpha dimension (α) and McDonald’s omega (Ω). Table 7 shows the satisfactory indices of internal consistency (DeVellis, 2003), since all of the dimensions exceeded the value of 0.7.

**TABLE 7:** Overall reliability of the questionnaire and reliability by dimensions.

Dimensions	Cronbach’s α	McDonald’s Ω
Overall	0.975	0.982
Communication with the school	0.731	0.703
Participation in school activities	0.875	0.840
Sense of belonging	0.915	0.899
Involvement in the home	0.921	0.904
Involvement in the PA and School Board	0.975	0.959
Community Participation	0.874	0.889
Training	0.916	0.816

All of the items complied with the corrected item-total correlation; thus it was not necessary to eliminate any of them, either in the QFIS-TP or in the dimensions making up the same. The dimension having the lowest reliability was that of family communication with the school, and the most consistent one was family involvement in the PA and School Board.

## Discussion

This study confirms that the psychometric quality of the QFIS-TP, adjusted to the theoretical approach of the IMFIS-TP, is satisfactory. So, the seven dimensions making it up explain a variance of 64.70%, having a high reliability, without modifications in the location of the items with respect to the model. However, the results of the CFA reveal that the octodimensional structure is best, dividing PA and the School Board into two dimensions, given that they refer to distinct representation organisms (García-Sanz et al., 2020).

The results of the structural equations model have offered covariance between measurement errors of some of the observable variables. However, while this covariance is inevitable, it may be considered appropriate (Landis et al., 2009). In this case, although the reduction of the affected items is similar, they are necessary to confirm the specified theoretical model.

So, based on the results obtained in each of the QFIS-TP subscales, it is evident that the “promoter of communication with families” and “promoter of sense of belonging” roles are theoretical and statistically related (Tran, 2014), and demonstrate a high internal consistency, although covariance has been registered between the measurement error of the variables, encourage meetings with other teachers (Q5), and defense of the school in competitions (Q19). On the other hand, the teacher’s role as “promoter of school activities” has produced diverse covariance between measurement errors, suggesting that for teachers, the actions are generalized, categorically hindering their definition. So, we believe that it is necessary to offer more specialized training with this respect, since studies clearly differentiate between classroom (Q7), cultural (Q8), sporting (Q9), and fund collection support (Q14) activities (Hornby, 2011; Epstein et al., 2019). Likewise, although work by commissions (Q13) as a resource may be useful in service (Q12) and school evaluation (Q15) activities, they have specifications of differentiated contents and they operate on different planes. In the first case, in the design of plans and in the following, in implementation and participation. However, Lingard et al. (2014) positively assessed family inclusion in work commissions for educational assessment and restructuring, since they promote active listening and multidirectional dialogue, despite teachers’ difficulties in taking advantage of the parents’ opinions.

As for “promoting involvement in the home,” covariance has been identified in the following measurement errors: Q28–Q29 and Q36–Q39. While positive parent-child relationships are sustained by communication and trust (Q28), this latter should not remain implicit, but rather, must be manifested (Q29) (Ebbert et al., 2019). On the other hand, a confusion between extracurricular (Q36) and cultural (Q39) activities has been confirmed, treating them as synonymous (Ladky and Peterson, 2008), when in fact, the former are academic and individualistic activities, while the latter are collective and are linked to family leisure (Hernández-Prados and Álvarez-Muñoz, 2019).

The subscale to promote the PA is not a generalized action of the teacher consisting of attending meetings (Garbacz et al., 2019), but, on the contrary, it consists of diverse aspects, since helping families getting familiarized with the PA as an organization (Q42) is different from informing them of activities offered to the families (Q44), just like encouraging the use of a banks of books (Q45) and checking the PA website (Q46). On the other hand, according to García-Sanz et al. (2020), the School Board is not as well-known as the PA and requires that teachers promote the knowledge of their representatives (Q52), but also, their organization and functioning (Q51) and the decisions that they make (Q53). Similar items have been proposed by Yulianti et al. (2019). Finally, the relationship between both dimensions reveals itself theoretically (Consejo Escolar del Estado, 2014; Epstein et al., 2019) in the covariance between measurement errors (Q45–Q51 and Q49–Q53), even though each was saturated in different factors.

Seventh, the teacher as “promoter of community participation” is recognized in educational policy, but has barely been developed in Spain, justifying the diversity of covariance between measurement errors. While neighborhood activities (Q60) may be ecological (Q59) or religious (Q62), according to Yulianti et al. (2019), these have been differentiated between, since they can be carried out by other associations. On the other hand, collection activities (Q58) are civic and solidarity-based, being external to the school environment, whereas textbook collection and distribution (Q45) is internal, and ecological activities (Q59) do not pursue collection, but rather, active involvement. All of these “are important with a strong range of institutions (religious, recreational, corporate, and library) that are linked to maintain stability, cohesion, and well-being of the community” (Gahwaji, 2019, p. 11).

Finally, “promoting family training” has covariance of measurement errors with variables from the previous subscale (Q64–Q71 and Q58–Q67), since according to IMFIS-TP, they are related to one another. In fact, taking advantage of free time (Q64) is one of the recurrent topics of the training offer (Q71) (Hernández-Prados and Álvarez-Muñoz, 2019).

The creation of this instrument can allow us to know the reality of the teacher and promote measures for training from the university (initial education), teacher training centers, and internal training promoted by the school (permanent training).

Based on all of this, the study has demonstrated that the techniques developed to assess the structural equations model have a confirmatory bias. Therefore, although the proposed model has an acceptable fit, the researcher has not tested it, but rather, has only confirmed that it is one of the various potential models (Cupani, 2012). As for the study’s limitations, although the QFIS-TP suitably fits the IMFIS-TP and permits knowledge of the level of competency of the teachers as promoters of family participation, future works should expand and diversify the sample of teachers, both in a national and international scope. Longitudinal studies are needed, in contrast with the qualitative studies, to identify other actions that teachers promote and that may not have been considered in the QFIS-TP. All of this will favor the creation of instruments that offer knowledge having a better fit with the teacher’s reality and will promote training measures from the university (initial education), the teacher training centers, and internal training promoted by the school. It would also be interesting to determine the teacher’s level of belonging to the school, since they cannot promote this if they themselves do not experience it. All of this suggests that this emerging field demands further research to help improve family participation.

## Data Availability Statement

The data are available and will soon appear in the Centro de Investigaciones Sociológicas (CIS) database called ARChivo de Estudios Sociales (ARCES) which can be accessed through http://www.arces.cis.es/arces.jsp.

## Author Contributions

MGO: exhaustive review of theoretical contributions, active collaboration in the construction of the questionnaire, general and specific proposals regarding the methodology, writing of the discussion section, and conclusions based on the results obtained. JP and MG-S: methodological development and analysis of results. MH-P: review for the theoretical basis, discussion, and conclusions. All authors contributed to the article and approved the submitted version.

## Conflict of Interest

The authors declare that the research was conducted in the absence of any commercial or financial relationships that could be construed as a potential conflict of interest.

## Publisher’s Note

All claims expressed in this article are solely those of the authors and do not necessarily represent those of their affiliated organizations, or those of the publisher, the editors and the reviewers. Any product that may be evaluated in this article, or claim that may be made by its manufacturer, is not guaranteed or endorsed by the publisher.
